# Development of real-time and lateral flow recombinase polymerase amplification assays for rapid detection of *Schistosoma mansoni*

**DOI:** 10.3389/fmicb.2022.1043596

**Published:** 2022-11-18

**Authors:** Silvia Gonçalves Mesquita, Elena Birgitta Lugli, Giovanni Matera, Cristina Toscano Fonseca, Roberta Lima Caldeira, Bonnie Webster

**Affiliations:** ^1^Grupo de Pesquisa em Helmintologia e Malacologia Médica, Instituto René Rachou, Fundação Oswaldo Cruz, Belo Horizonte, Brazil; ^2^Wolfson Wellcome Laboratories, Department of Science, Natural History Museum, London, United Kingdom; ^3^Department of Health Sciences, Unit of Microbiology, University “Magna Graecia” of Catanzaro, Catanzaro, Italy; ^4^Grupo de Pesquisa em Biologia e Imunologia Parasitária, Instituto René Rachou, Fundação Oswaldo Cruz, Belo Horizonte, Brazil

**Keywords:** Schistosomiasis, *Schistosoma mansoni*, Recombinase Polymerase Amplification, isothermal molecular diagnostics, mitochondrial minisatellite region, real-time RPA, lateral flow RPA

## Abstract

**Background:**

Accurate diagnosis followed by timely treatment is an effective strategy for the prevention of complications together with reducing schistosomiasis transmission. Recombinase Polymerase Amplification (RPA) is a simple, rapid, sensitive, and specific isothermal method with low resource needs. This research aimed at the development and optimisation of a real-time (RT) and a lateral flow (LF) RPA assay for the detection of *Schistosoma mansoni*.

**Methodology:**

Recombinase Polymerase Amplification reactions were performed at full- (as recommended) and half-volumes (to reduce costs), with RT or LF detection systems targeting the *S*. *mansoni* mitochondrial minisatellite region. The specificity was assessed using gDNA from other *Schistosoma* species, helminths co-endemic with *S*. *mansoni*, human stool, and urine, and *Biomphalaria* snail hosts. The analytical sensitivity was evaluated using serial dilutions of gDNA, synthetic copies of the target, and single eggs. The ability of both assays to detect the *S*. *mansoni* DNA in human urine and stool samples was also tested. The long-term stability of the RT-RPA reagents was evaluated by storing the reaction components in different temperature conditions for up to 3  weeks.

**Results:**

The RT- and the LF-RPA (SmMIT- and SmMIT-LF-RPA, respectively) presented similar results when used full- and half-volumes, thus the latter was followed in all experiments. The SmMIT-RPA was 100% specific to *S*. *mansoni*, able to detect a single egg, with a limit of detection (LOD) of down to 1  fg of gDNA and one synthetic copy of the target. The assay was able to detect *S*. *mansoni* DNA from stool containing 1 egg/g and in spiked urine at a concentration of 10  fg/μl. SmMIT-RPA reagents were stable for up to 3  weeks when kept at 19°C, and 2  weeks when stored at 27°C. The SmMIT-LF-RPA cross-reacted with Clinostomidae, presented the LOD of 10  fg and one synthetic copy of the target, being able to detect a single egg and 1 egg/g in a stool sample. The LOD in spiked urine samples was 10  pg/μl.

**Conclusion:**

The half-volume SmMIT-RPA is a promising method to be used in the field. It is specific, sensitive, robust, and tolerant to inhibitors, with a long-term stability of the reaction components and the real-time visualisation of results.

## Introduction

Schistosomiasis is a neglected tropical disease associated with poverty and low sanitation conditions, causing more than 240 million cases worldwide and 1.4 million disability-adjusted life years (DALYs). It is estimated that 779 million people currently live at risk of infection in tropical and subtropical regions (Kyu et al., 2018; Panzner, 2022; World Health Organization, 2022). The clinical manifestations of the disease can be urogenital or intestinal depending on the species that is causing the infection. *Schistosoma mansoni* is the species that causes intestinal disease in Africa and the Americas. The infection occurs when people have contact with watercourses contaminated with cercariae shed by *Biomphalaria* snails. The cercariae actively penetrate human skin, losing their tail, and migrating as schistosomula *via* blood vessels until establishing infection within the mesenteric veins as mature adult worms. After copulation, the female worm laid up to 300 eggs per day that can be released into the environment through the host’s faeces or become trapped in tissues, causing most of the chronic symptoms and complications of the disease (McManus et al., 2018; LoVerde, 2019; Nelwan, 2019). Nonspecific symptoms may occur in the early stage of the infection, such as fever, headache, fatigue, and myalgia and it is known as Katayama syndrome. Chronic schistosomiasis often produces gastrointestinal symptoms including diarrhea and abdominal pain, as well as hepatosplenic symptoms due to eggs lodged in the liver, e.g., fibrosis and portal hypertension (Colley et al., 2014). Less commonly, complications associated with ectopic migration of eggs can be observed in the brain and spinal cord (Vale et al., 2012).

The WHO Guidelines on Control and Elimination of Human Schistosomiasis were recently published. WASH (clean water, sanitation, and hygiene) and environmental interventions are highly recommended together with preventive chemotherapy (PC) by the mass drug administration (MDA) of Praziquantel targeting selected areas and groups (Lo et al., 2022; World Health Organization, 2022). MDA success is conditional to the precise assessment of schistosomiasis prevalence that will determine the appropriate strategy to be used (Utzinger et al., 2015). Therefore, estimating the true prevalence of schistosomiasis has a significant impact on the control and elimination measures (Turner et al., 2017).

The Kato-Katz (KK) technique is the method recommended by the WHO for the diagnosis of human intestinal schistosomiasis and it consists of the microscopic visualisation of eggs in the stool. This test is highly specific, cost-effective, and simple to perform, not needing much technological equipment other than the optic microscope (Katz et al., 1972). It has been extensively used for epidemiological surveys presenting a satisfactory performance in high prevalence settings. However, the KK’s sensitivity varies depending on the period of infection, daily fluctuation of egg excretion, uneven distribution of eggs in the stool, endemicity, and/or co-endemicity of the area (Bärenbold et al., 2017; Cavalcanti et al., 2019; Diego et al., 2021; Ogongo et al., 2022). These limitations are mainly observed in moderate and low endemic areas, where 25–30% of positive cases can be missed (Berhe et al., 2004; Enk et al., 2008; McManus et al., 2018). Schistosomiasis prevalence and intensity of infection has decreased in many endemic regions over the past years, especially due to MDA and WASH improvements (Katz, 2018; Brasil, 2021; Lo et al., 2022). Since the occurrence of light infections is becoming more frequent, the development and implementation of new diagnostic tools are highly needed (Utzinger et al., 2015).

Antigen tests based on the detection of circulating anodic and cathodic antigens (CAA and CCA, respectively) can be used for the indirect detection of *S*. *mansoni* using urine and serum samples. The detection of antigens can be performed using two types of lateral flow assays named POC-CCA (commercially available) and UCP-LF-CAA. Both assays are more sensitive than KK, in particular, the UCP-LF-CAA, which is the most sensitive and specific antigen test currently available (Sousa et al., 2019; Assare et al., 2021). However, there are limitations related to these tests. CCA detection does not work for urogenital schistosomiasis. It has been shown to give false-positive results (Graeff-Teixeira et al., 2021) with performance issues recently reported related to different kit batches (Viana et al., 2019) and with complicated interpretation of trace results (Coelho et al., 2016). Although the UCP-LF-CAA assay is very promising and covers all *Schistosoma* species (Corstjens et al., 2014), it currently needs bespoke laboratory based equipment with 24 h needed to obtain results (Sousa et al., 2019).

Molecular PCR-based methods have been extensively used for schistosomiasis detection due to the high sensitivity, specificity, and accuracy when compared to the KK technique (Pontes et al., 2003; Gomes et al., 2006, 2010; Cnops et al., 2012; Meurs et al., 2015; Frickmann et al., 2021; Siqueira et al., 2021). Despite having great advantages, the use of PCR-based methods is limited by the elevated cost and the need for advanced technological equipment and laboratory infrastructure, hampering large-scale implementation in endemic settings (Diego et al., 2021; Panzner, 2022).

Isothermal molecular methods stand out as promising alternatives to PCR for use at the point-of-care (POC)/point-of-need (PON). The loop-mediated isothermal amplification (LAMP) and the recombinase polymerase amplification (RPA) are the most common approaches used, providing fast and sensitive diagnosis and feasible in the field as they have low resource needs (Lobato and O’Sullivan, 2018; Li et al., 2021). Several LAMP assays have been developed for the detection of *S*. *mansoni* in both human and snail hosts over the past years, with favorable results (Abbasi et al., 2010; Hamburger et al., 2013; Fernández-Soto et al., 2014; Gandasegui et al., 2016, 2018; Caldeira et al., 2017; Mwangi et al., 2018; García-Bernalt Diego et al., 2019; Price et al., 2019; Mesquita et al., 2021). RPA was described in 2006 (Piepenburg et al., 2006) and since then it has been used mostly for the detection of *Schistosoma haematobium* (Rosser et al., 2015; Rostron et al., 2019; Archer et al., 2020, 2022; Frimpong et al., 2021) and *Schistosoma japonicum* (Sun et al., 2016; Xing et al., 2017; Guo et al., 2021; Deng et al., 2022), with only one study focused on *S*. *mansoni* targeting the ribosomal DNA (rDNA) regions 28S and the internal transcribed spacer (ITS; Poulton and Webster, 2018). Although this work represented a first and important step for the use of RPA for *S*. *mansoni* diagnosis, the lateral flow approaches lacked specificity with cross-reactivity with *S*. *haematobium* and *Schistosoma bovis* observed. One of the benefits of molecular based approaches is that they can be designed for different DNA biomarkers, allowing assays to be optimised to achieve high levels of sensitivity and specificity (Wang and Hu, 2014; Blasco-Costa et al., 2016). Once the isothermal molecular platform, such as LAMP and RPA, has been established and proved to work in the required settings then the molecular assays can be tailored to the need and sample type. This versatility of molecular platforms presents many cross-cutting opportunities and financial value.

*Schistosoma mansoni* molecular detection generally relies on stool samples, but urine, serum, and saliva can also be used for that purpose due to the presence of cell-free DNA (cfDNA; LoVerde, 2019). Each type of sample used as a source for DNA has its particularities. Stool samples are widely utilised as the source of both cfDNA and DNA from eggs, the latter often attached to a bead-beating and/or freezing step to facilitate egg disruption and DNA release (Pomari et al., 2019; Barda et al., 2020). Despite stool samples being non-invasive, they are inconvenient and require community sensitisation to ensure the collection of samples (Turner et al., 2017). Conversely, cfDNA presents great advantages as some bodily fluids such as urine are non-invasive, convenient, and usually easier to process, not requiring additional steps for sample preparation (Weerakoon and McManus, 2016). Sample type and sample preparation also have an effect on the downstream molecular assay to be used/tested. For example, PCR based approaches typically need samples that have been processed to remove inhibitors while isothermal assays are more tolerant to such inhibitors (Lobato and O’Sullivan, 2018). However, sample preparation and DNA extraction are currently among the factors that limit the use of molecular-based diagnostics in resource-poor settings and at the POC/PON due to the equipment requirements, costs, and time needs.

Moving from morbidity control to elimination as a public health problem requires more sensitive and specific diagnostic tests, especially to verify interruption of transmission, by detecting the infection in humans and snails (World Health Organization, 2022). Generally, as the demand for the novel test increase, its cost tends to decrease. Also, the cost-effectiveness of more accurate tests usually outweigh the actual cost of the test and the economic cost of the disease (Turner et al., 2017). For instance, it is estimated that in Brazil schistosomiasis generates annually a financial burden of nearly 41 million USD. More than 90% of that is related to indirect costs (e.g., loss of productivity and wages due to sick leave, hospitalisation, and premature deaths) that could be avoided by accurate diagnosis of infected people and timely treatment (Nascimento et al., 2019).

In this research, we developed and evaluated the performance of a real-time and a lateral flow RPA assay targeting the mitochondrial minisatellite region of *S*. *mansoni* to evaluate the diagnosis of the infection in humans and snails, especially in endemic areas where resources are limited.

## Materials and methods

### Samples used for assay development and optimisation

For analytical sensitivity and specificity testing, genomic DNA (gDNA) from *S*. *mansoni* and other *Schistosoma* species (*Schistosoma haematobium, Schistosoma curassoni,* and *Schistosoma bovis*) were obtained from the Schistosomiasis Collection at The Natural History Museum (SCAN; Emery et al., 2012). Further analytical specificity was evaluated using gDNA from other non-*Schistosoma* samples including the intermediate hosts *Biomphalaria glabrata, Biomphalaria tenagophila*, *Biomphalaria straminea,* and trematodes commonly found infecting *Biomphalaria* snails belonging to the families Clinostomidae, Echinostomatidae, Notocotylidae, Spirorchiidae, and Strigeidae, all obtained from the Medical Malacology Collection at René Rachou Institute, Fiocruz Minas (Fiocruz-CMM) via the Trematodes Biology Laboratory from the Federal University of Minas Gerais (UFMG). The Helminthology and Medical Malacology Laboratory (HMM) from Fiocruz Minas provided gDNA from helminths co-endemic with *S*. *mansoni* including *Ascaris lumbricoides,* Ancylostomidae*, Enterobius vermicularis, Trichuris trichiura*, and *Fasciola hepatica*. Clinical stool samples were obtained under the Ethical Committee of Calabria Region approval (#108, 27 April 2017) and provided by the University “Magna Graecia” of Catanzaro. The data associated to all the specimens used in this study is provided on Supplementary material 1.

### RPA primers and probe design

**Figure 1:**
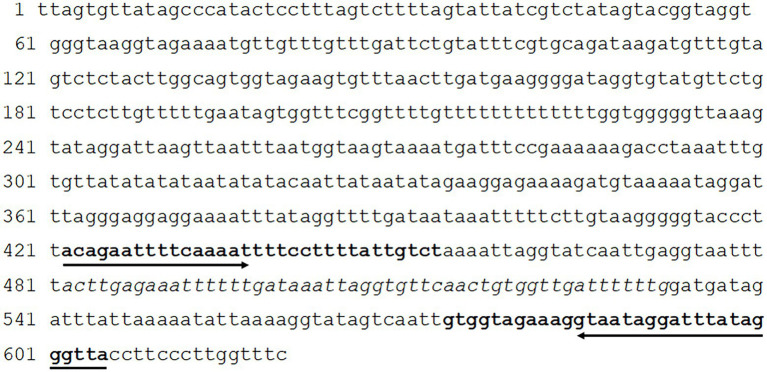
Position of the Recombinase Polymerase Amplification (RPA) primers and probe within the Mitochondrion *Schistosoma mansoni* minisatellite DNA region. Bold sequences represent the primer sites and sequence in italics represents the probe site.

Recombinase Polymerase Amplification primers and the internal probe (TIB MolBio-Berlin, Germany) were designed targeting the *S*. *mansoni* mitochondrial minisatellite DNA region (GenBank accession number: L27240) following the guidelines from TwistDx™ (Cambridge, United Kingdom). To prevent the formation of primer-dimers, a phosphothioate backbone was added to the reverse primers for both the LF and RT assays (El Wahed et al., 2021) and the position of the 6-FAM and BHQ1 was reversed (compared to the design guidelines) within the RT probe. The primers were tested in-silico using BLAST (Altschul et al., 1990) to check the possibility of cross-reactivity. All primers and probes are described in Table 1 and shown in Figure 1.

**Table 1:** Mitochondrial primer and probe sequences designed for the LF-and RT-RPA.

Name	RPA method	Sequence (5′-3′)
SmMITnfo probe	LF	(6-FAM)ACTTGAGAAATTTTTTGATAAATTAGGTGTTC(THF)ACTGTGGTTGATTTTTTG(c3)
SmMITnfo reverse	LF	(Btn)TAACCCTATAAATCCTATTACCTTTCTACCAsC
SmMIT forward	LF/RT	ACAGAATTTTCAAAATTTTCCTTTTATTGTCT
SmMIT probe	RT	ACTTGAGAAATTTTTTGATAAATTAGGTGT(BHQ1)C(THF)AC(6-FAM)GTGGTTGATTTTTTG(c3)
SmMIT reverse	RT	TAACCCTATAAATCCTATTACCTTTCTACCAsC

6-FAM, 6-carboxyfluorescein; THF, tetrahydrofuran residue; BHQ1, black hole quencher; s, thiol group; c3, C3 Spacer; Btn, Biotin; LF, lateral flow; and RT, real time.

### RPA reaction setup

#### Real-time fluorescence-based RPA (SmMIT-RPA)

**Figure 2:**
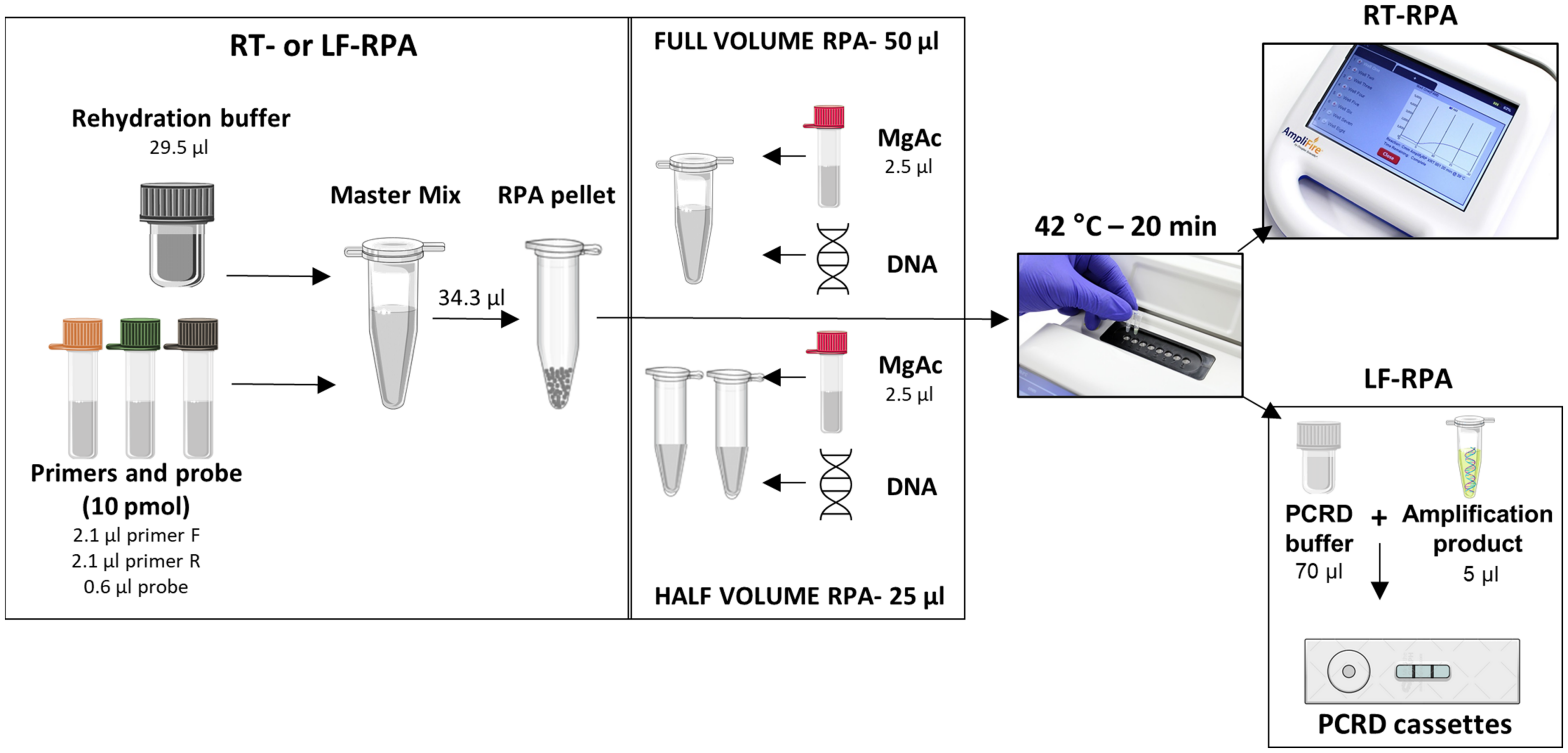
Set up of each RPA protocol used in this study. Images used in this figure were obtained at Agdia, Bioicons, and Mind the Graph websites.

The RT SmMIT-RPA was performed using the TwistAmp® Exo Kit (TwistDX, Cambridge, United Kingdom). RPA reactions were set up as recommended by the manufacturer. Reactions were run in volumes of 50 μl (as recommended) or 25 μl (half-reactions; Supplementary material 2A). For the 50 μl reaction, a master mix was prepared containing the RPA rehydration buffer, water (if needed), forward and reverse primers, and the RT probe. This was then added to the lyophilised RPA pellet and homogenised by pipetting. The magnesium acetate (MgAc) was added to the lid of the RPA tube. Lastly, the DNA was added to the reaction tube. For the 25 μl reaction, a master mix was prepared and added to the lyophilised RPA pellet as above, and once homogenised it was split into two new tubes. The MgAc was added to the lid of each tube and finally, the DNA was added to the reaction. A positive (1 ng of *S*. *mansoni* gDNA) and negative (water) controls were included in all the runs. For both protocols, after the DNA addition, the tubes were quickly (~2 s) centrifuged, mixed (by inversion), then quickly (~2 s) centrifuged again. The centrifugation step mixes the MgAc with the other components which starts the RPA reaction. The tubes were incubated at 42°C for 20 min using the portable fluorometer AmpliFire (Douglas Scientific, Alexandria, MN, United States), with a manual mix after 4 min of incubation. Results could be seen in real-time *via* the device’s touchscreen and exported as an excel file for analysis of the raw data. Samples were considered positive if the amplification curve, normalised by the background level during the initial 4 min of the reaction, crossed the threshold of 346 relative fluorescence units (RFU). To determine the threshold value, we calculated the mean RFU observed in the first 4 min of the reaction of all non-*S*. *mansoni* samples used during the standardisation phase. The threshold was then set by three standard deviations of the calculated means, which was 346 RFU. All the protocol steps and the amount of each reagent are detailed in Figure 2 and Supplementary material 2A.

#### Lateral flow RPA (SmMIT-LF-RPA)

The SmMIT-LF-RPA was performed using the TwistAmp® Nfo Kit (TwistDX, Cambridge, United Kingdom). The reaction was set up as described above and detailed in Figure 2 and Supplementary material 2B. A positive (1 ng of *S*. *mansoni* gDNA) and negative (water) controls were included in all the runs. After incubation, 5 μl of the amplification product were added to a 0.5 ml tube together with 70 μl of the PCRD extraction buffer. The mixture was then added to PCRD lateral flow cassettes. Results were observed after 10 min and any changes in the results after that period were not acknowledged. Positive samples presented two lines (both test and control lines) while just the control line was present for negative samples.

### RPA limit of detection

The LOD of the assays was evaluated using the samples as described below.

#### Genomic DNA and synthetic copies

Serial 10-fold dilutions from 1 ng to 1 fg of *S*. *mansoni* gDNA were prepared from an adult worm extracted using the DNeasy Blood & Tissue kit (Qiagen, Hilden, Germany) at an original concentration of 3.8 ng/μl, measured by Nanodrop Spectrophotometer (ThermoFisher, Massachusetts, United States). Synthetic copies of the target region were obtained (gBlocks, IDT, Newark, New Jersey, United States) and diluted from 1 × 10^5^ copies/μl down to 1 copy/μl. The analytical sensitivity of SmMIT-RPA was calculated by repeating 10 times the reaction using gDNA dilutions and three times using synthetic copies dilutions.

#### *Schistosoma mansoni* eggs

Single eggs of *S*. *mansoni* were provided by the Snail Schistosome Resource (SSR, Natural History Museum, United Kingdom-https://www.nhm.ac.uk/our-science/our-work/sustainability/schistosome-snail-resource.html) *via* the NIAID Schistosomiasis Resource Center (SRC, Biomedical Research Institute, United States-https://www.afbr-bri.org/schistosomiasis/). Individual eggs were isolated from a pool of eggs by capturing each one with a micropipette under a stereomicroscope. The DNA was then extracted using the SwiftX™ DNA kit (Xpedite Diagnostics, Germany) following protocol 1 from the manufacturer, with and without the heating step (Supplementary material 3). In brief, the protocol consists of the addition of buffer DL (50 μl) and magnetic beads (7.5 μl) to the tubes containing the single egg and incubate at room temperature (~19°C) or heated (95°C) for 5 min, followed by magnetic separation of the supernatant, which contains the extracted DNA. The direct addition of whole single eggs, with no DNA extraction procedure, in fresh (live eggs) and frozen conditions was also tested.

### RPA specificity

For both the LF and RT assays, the specificity was assessed against gDNA from other organisms that may be present in clinical and field samples. This included host DNA (snail host DNA and DNA from human urine and stool). gDNA from the snail hosts *B*. *glabrata, B*. *straminea*, and *B*. *tenagophila* were tested together with other trematodes that are commonly found infecting *Biomphalaria* snails in the neotropical region (Clinostomidae, Echinostomatidae, Notocotylidae, Spirorchiidae, and Strigeidae). Helminths of medical importance that are often co-endemic with *S*. *mansoni* (*A*. *lumbricoides,* Ancylostomidae*, E*. *vermicularis, F*. *hepatica,* and *T*. *trichiura*) were tested together with other commonly occurring *Schistosoma* species (*S*. *bovis, S*. *curassoni, S*. *haematobium*).

### RPA performance with urine and stool

#### Clinical stool samples

Stool samples that had been previously collected and characterised were used. One sample collected in Guinea Bissau, negative by both Kato-Katz and PCR methods, and one sample from Colombia, positive by Kato-Katz (1 egg/g) and by PCR. gDNA from these samples was extracted using the QIAamp DNA Stool Mini Kit (Qiagen, Hilden, Germany) following the manufacturer’s protocol.

#### Spiked urine samples

Seven aliquots of 100 μl of urine from a non-infected donor were spiked with different concentrations of *S*. *mansoni* gDNA with final concentrations within the samples ranging between 1 ng/μl and 1 fg/μl. Two aliquots of 100 μl of urine from the same donor were spiked with a pool of gDNA from medically important helminths (*A*. *lumbricoides,* Ancylostomidae*, E*. *vermicularis, F*. *hepatica,* and *T*. *trichiura*), with and without *S*. *mansoni* DNA. One aliquot of 100 μl of urine without the addition of any DNA was also used. The urine samples were filtered using Whatman® qualitative filters paper grade 3:6 μm (Sigma-Aldrich, St. Louis, Missouri, United States). The filters were dried at room temperature and 6 mm holes were made using a hole puncher. The paper holes were used for the DNA extraction using the QIAamp DNA Blood Mini Kit (Qiagen, Hilden, Germany) and following the protocol described by Lodh et al. (2017).

### SmMIT-RPA storage conditions

Aiming to simulate point-of-need settings, we evaluated alternative storage conditions of the RT primers, probe, and kit reagents for up to 3 weeks. The reaction components were stored protected from light, at 19 and 27°C, mixed or separate. Reactions were conducted on days 0, 1, 2, and 3, and on weeks 1, 2, and 3, so we could evaluate the efficiency of the reaction over time.

## Results

### Primers and probes

Primers and probes targeting 184 bp of the mitochondrial *S*. *mansoni* minisatellite DNA region were manually designed as detailed in Table 1. In-silico specificity, based on BLAST (Altschul et al., 1990), was 100% specific for *S mansoni*.

### SmMIT-RPA and SmMIT-LF-RPA assay testing

Both real-time and lateral flow assays were successfully performed using full (50 μl) and half (25 μl) reaction volumes, keeping the final concentrations of each reaction component as in the original protocol (Table 2; Supplementary material 4). For that reason, the adapted protocol was followed in all experiments performed and the results presented from here on in were obtained by using half reaction volumes (25 μl), as detailed in Figure 2.

**Table 2:** The performance of SmMIT-and SmMIT-LF-RPA assays.

Tests		SmMIT-RPA	SmMIT-LF-RPA
**Volume**	Full	Good performance	Good performance
	Half	Good performance	Good performance
**Specificity**	*Schistosoma* species	No cross-reactivity	No cross-reactivity
	Trematodes	No cross-reactivity	Cross-reactivity with Clinostomidae
	Helminths	No cross-reactivity	No cross-reactivity
	*Biomphalaria* snail hosts	No cross-reactivity	No cross-reactivity
	Human urine and stool	No cross-reactivity	No cross-reactivity
**Analytical Limit of Detection**	gDNA	1 fg	10 fg
	Synthetic copies	1 copy	1 copy
	Single Egg	Positive	Positive
**Laboratory Validation**	Stool	1egg/g	1 egg/g
	Urine	10 fg/μl	10 pg./μl
**Storage**	Primer mix+ Probe at 19°C	Stable until week 3	Not evaluated
	Primers separate + Probe at 19°C	Stable until week 3	
	Kit at 19°C	Stable until week 3	
	Primer mix+ Probe at 27°C	Stable until week 2	
	Primers separate + Probe at 27°C	Stable until week 2	
	Kit at 27°C	Stable until week 3	

### Real-time fluorescence-based RPA (SmMIT-RPA)

#### SmMIT-RPA limit of detection

**Figure 3:**
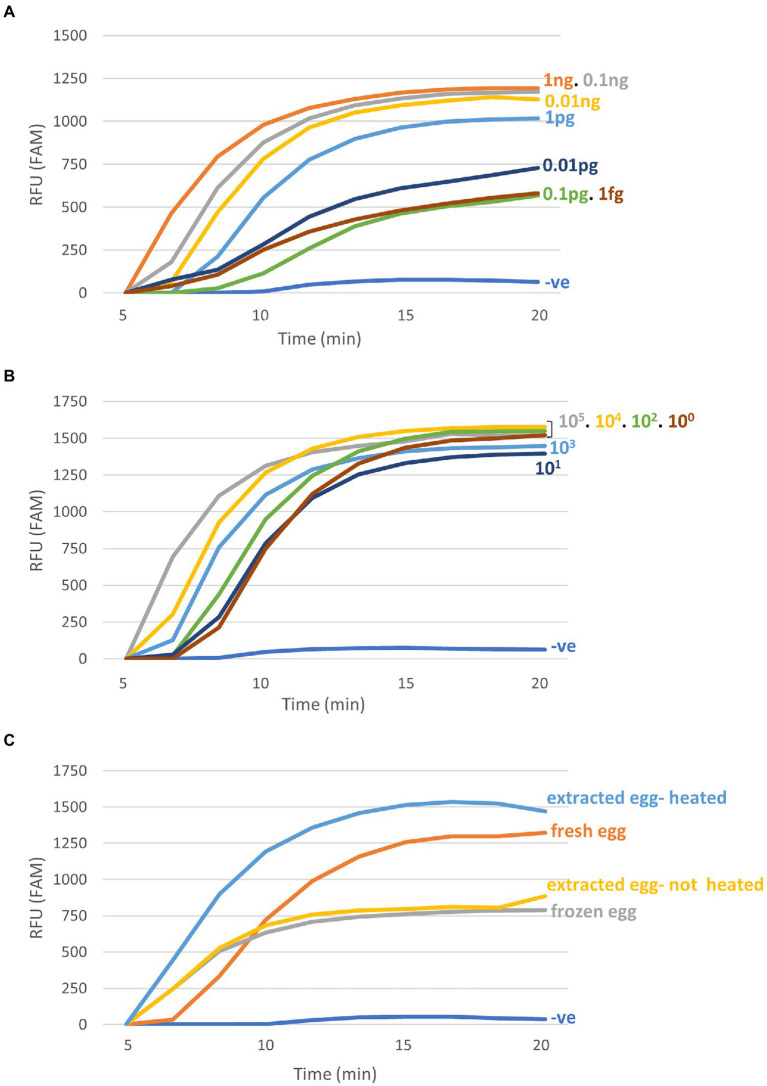
Limit of detection of the SmMIT-RPA. The assay was able to detect down to **(A)** 1  fg of the gDNA; **(B)** One synthetic copy of the target; **(C)** Single eggs extracted with the SwiftX™ DNA kit with or without the heating step, and single crude eggs, collected fresh and frozen (no DNA extraction procedure used). All graphs are displayed with the background baseline fluorescence subtracted. Legend: RFU-relative fluorescence units; ng-nanogram; pg-picogram, fg-femtogram; −ve-negative control (water).

The SmMIT-RPA presented a high limit of detection being able to detect down to 1 fg of *S*. *mansoni* gDNA, one synthetic copy of the target, and a single *S*. *mansoni* egg in all conditions evaluated being: (i) DNA extracted with heated incubation; (ii) DNA extracted without heated incubation; (iii) fresh non-extracted egg; and (iv) frozen non-extracted egg (Figure 3; Table 2).

#### SmMIT-RPA specificity

**Figure 4:**
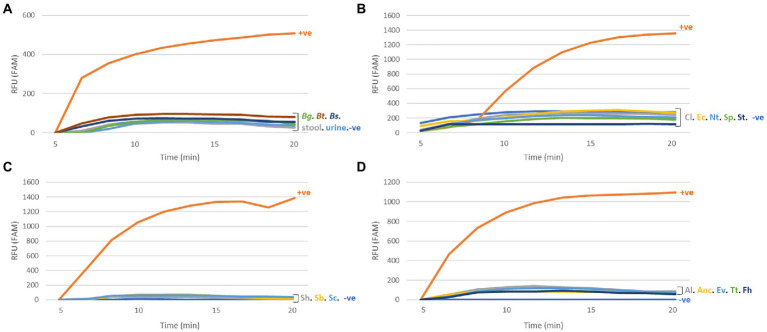
Specificity of the SmMIT-RPA. The assay was specific to *Schistosoma mansoni* with no cross-reactivity against **(A)** The host species; **(B)** Trematodes; **(C)** *Schistosoma* species; **(D)** Helminths of medical importance. All graphs are displayed with the background baseline fluorescence subtracted. Legend: RFU-relative fluorescence units; +ve-positive control (1  ng of *S*. *mansoni* gDNA); −ve-negative control (water); Bg, *B*. *glabrata*; Bt, *B*. *tenagophila*; Bs, *B*. *straminea*; Cl, Clinostomidae; Ec, Echinostomatidae; Nt, Notocotylidae; Sp, Spirorchiidae; St, Strigeidae; Sh, *S*. *haematobium*; Sb, *S*. *bovis*; Sc, *S*. *curassoni*; Al, *A*. *lumbricoides*; Anc, Ancylostomidae; Ev, *E*. *vermicularis*; Fh, *F*. *hepatica*; and Tt, *T*. *trichiura*.

The SmMIT-RPA assay was specific to *S*. *mansoni* with no cross-reactivity observed with snail or human DNA, DNA from other trematodes (including other *Schistosoma* species), and other helminths of medical importance (Figure 4; Table 2).

#### SmMIT-RPA performance using spiked urine and stool samples

The SmMIT-RPA presented a good performance when used on biological samples. *Schistosoma mansoni* DNA was detected in the positive stool sample known to contain 1 egg/g of stool (previously detected by the KK technique), and urine samples spiked with *S*. *mansoni* DNA at a final concentration of 10 fg/μl (Table 2; Supplementary material 5A) indicating high tolerance to molecular assay inhibitors present in urine (e.g., urea).

#### SmMIT-RPA storage condition

Our results showed that storing the primers and probe mixed together affected the assay performance giving false positive results. Keeping just the two primers mixed together or separately did not affect the RPA performance. However, the storage temperature influenced the results. Storing the primers and probe at 27°C reduced their longevity to 2 weeks, 1 week less than when stored at 19°C. The lyophilised RPA pellet, rehydration buffer, and MgAc produced consistent results after being stored for 3 weeks at both temperature conditions (19 and 27°C; Table 2). These results indicate that the SmMIT-RPA is a promising method able to produce robust results without the need for consistent cold chain for up to 3 weeks.

### Lateral flow RPA (SmMIT-LF-RPA)

#### SmMIT-LF-RPA limit of detection

**Figure 5:**
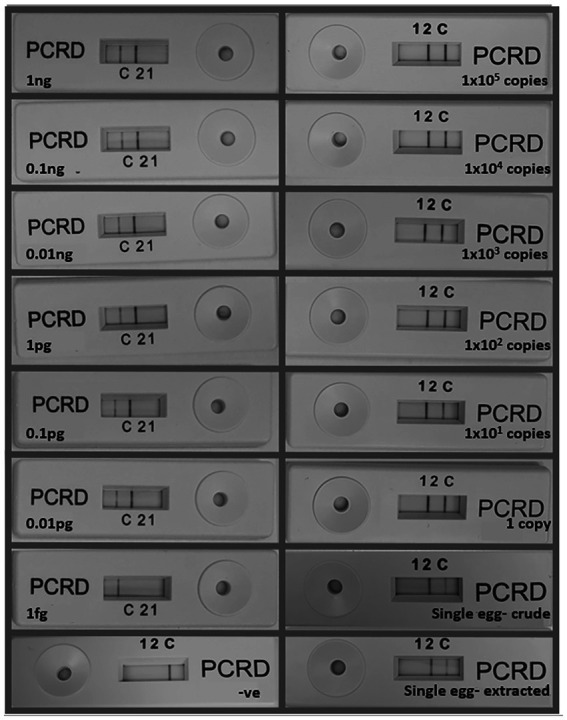
Limit of detection of the SmMIT-LF-RPA. The assay was able to detect down to 10  fg of the gDNA, one copy of the synthetic target DNA, and one single egg with or without DNA extraction. Legend: ng, nanogram; pg, picogram, fg, femtogram; +ve, positive control (1 ng of *Schistosoma mansoni* gDNA); and −ve, negative control (water).

The SmMIT-LF-RPA detected down to one synthetic copy of the target region and one single egg either crude (frozen) or extracted with the SwiftX™ DNA kit (original protocol, i.e., with the heating step). The limit of detection of the assay using serial dilutions of the gDNA was 10 fg, 10-fold less sensitive than the SmMIT-RPA (Figure 5; Table 2).

#### SmMIT-LF-RPA specificity

**Figure 6:**
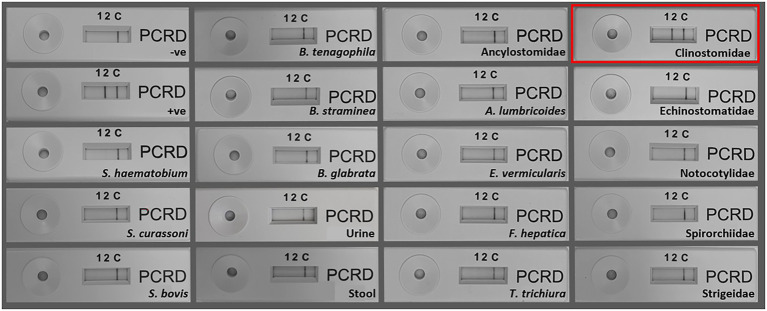
Specificity of the SmMIT-LF-RPA. The assay was specific to *Schistosoma mansoni* with no cross-reactivity with *S*. *haematobium, S*. *bovis, S*. *curassoni*, human stool, human urine *B*. *glabrata, B*. *straminea, B*. *tenagophila*, *A*. *lumbricoides,* Ancylostomidae, *E*. *vermicularis, T*. *trichiura, F*. *hepatica*, and trematodes belonging to the families, Echinostomatidae, Notocotylidae, Spirorchiidae, and Strigeidae. Cross-reactivity was observed for a Clinostomidae trematode (highlighted in red). Legend: +ve-positive control (1  ng of *S*. *mansoni* gDNA); −ve-negative control (water).

The SmMIT-LF-RPA assay showed high specificity for *S*. *mansoni* with no cross-reactivity against *B*. *glabrata, B*. *straminea*, *B*. *tenagophila*, human urine, and stool; nor was there cross-reactivity to other co-endemic human helminths and other trematodes belonging to the families Echinostomatidae, Notocotylidae, Spirorchiidae, Strigeidae, and three other *Schistosoma* species. However, cross-reactivity was observed with a trematode from the Clinostomidae family (Figure 6; Table 2).

#### SmMIT-LF-RPA performance using urine and stool samples

A loss in LF sensitivity was observed when spiked urine samples were analysed. SmMIT-LF-RPA was able to detect 10 pg/μl of *S*. *mansoni* DNA, a 1,000-fold decrease when compared to the SmMIT-RPA. The assay was able to detect the presence of the *S*. *mansoni* DNA in the positive stool sample (1 egg/g by the KK technique; Table 2; Supplementary material 5B).

## Discussion

Sensitive, specific, simple, and rapid diagnostic methods are essential to reach the World Health Organization (WHO) target of eliminating schistosomiasis as a public health problem (prevalence of heavy infections lower than 1%) by 2030 (WHO World Health Organization, 2022). To this end, Recombinase Polymerase Amplification (RPA) is an isothermal amplification method that presents great potential. It has been piloted for detection of urogenital schistosomiasis caused by *Schistosoma haematobium* (Rosser et al., 2015; Rostron et al., 2019; Archer et al., 2020; Frimpong et al., 2021) and intestinal schistosomiasis caused by *Schistosoma japonicum* (Sun et al., 2016; Xing et al., 2017; Guo et al., 2021; Deng et al., 2022), with promising results for its use in the field at the point-of-need (PON). Only one study has explored the development of a RPA assay to detect *Schistosoma mansoni* DNA, and this was done using the lateral flow RPA. The assay’s molecular targets were the 28S and ITS rDNA regions and although analytical sensitivity was good, the assays were found to be non-specific to *S*. *mansoni* with cross-reactivity observed with other *Schistosoma* species, namely *S*. *haematobium* and *S*. *bovis* (Poulton and Webster, 2018). The development of a RT-RPA targeting these ribosomal regions may be advantageous for a genus-specific assay. The genus or species level diagnostic specificity need for schistosomiasis, will depend on the diagnostic use case, with species level specificity detailed as a priority within the WHO schistosomiasis diagnostic target product profile (World Health Organization, 2021). In the present study, real-time fluorescence-based (RT) and lateral flow (LF) RPA assays were developed targeting the mitochondrial minisatellite region (termed here as SmMIT-and SmMIT-LF-RPA, respectively RPA) with higher sensitivity and specificity to *S*. *mansoni*, compared to the assays developed by Poulton and Webster (2018), with further potential for its use in endemic settings.

The developed SmMIT-RPA assay was 100% specific for *S*. *mansoni* in this study, proving to be a good alternative for the detection of *S*. *mansoni* infections in both human and snail hosts, without any cross-reactivity with other *Schistosoma* species and trematodes tested. It will be important to further test this specificity in terms of the genetic diversity of *S*. *mansoni* geographical strains, to make sure that specificity does not limit its ability to detect *S*. *mansoni* from all endemic areas (Webster et al., 2013). This is particularly relevant as the mitochondrial minisatellite target, used here, may be prone to high mutation rates due to its mitochondrial origins. It would also be relevant to check for specificity and cross-reactivity within co-infections, where inter-species interactions can lead to the production of hybrid offspring, e.g., *S*. *mansoni/S*. *haematobium* parthenogenic hybrids that have been observed to occur in co-endemic areas (Huyse et al., 2013). As the target used here is a mitochondrial target, only the maternal line of the interactions will be detected, however such hybrids are never found without the presence of the original species and so a specific diagnostic, such as the SmMIT-RPA, will still provide a diagnosis. The SmMIT-RPA assay should be further tested on *Schistosoma rodhaini,* the sister species to *S*. *mansoni* to check its ability to distinguish between *S*. *mansoni, S*. *rodhaini* and *S*. *mansoni/S*. *rodhaini* hybrids. This is particularly important for snail xenomonitoring of *Biomphalaria* snails, as *S*. *rodhaini* and *S*. *mansoni/S*. *rodhaini* are not implicated in human infections (Rey et al., 2021).

It was expected that the SmMIT-LF-RPA would show the same specificity as the primers and probes designed for both assays were very similar, except for the specific modifications required for each approach. However, cross-reactivity with cercariae belonging to the Clinostomidae family was observed only in the SmMIT-LF-RPA. Trematodes in the family Clinostomidae belong to the superfamily Schistosomatoidea together with the family Schistosomatidae, to which *S*. *mansoni* belongs. The close phylogenetic relationship between both families may reflect genomic similarities. RPA is known to be highly specific, but it can also tolerate the presence of a few nucleotide mismatches within the primer and probe regions, which can lead to false-positive results (Lobato and O’Sullivan, 2018), hence the need for rigorous specificity testing. Currently, there is no data available in public databases for the mitochondrial minisatellite region of non-schistosome trematodes, limiting the in-silico evaluation of the primers’ specificity. Species belonging to the Clinostomidae family have been reported parasitising *Biomphalaria* snails in Brazil, including the hosts species (*B*. *glabrata, B*. *straminea,* and *B*. *tenagophila*; Mesquita et al, 2020; Sousa et al., 2022). These trematodes are parasites of birds with fish being the second intermediate host. Human infections are rare and accidental due to the ingestion of raw fish. These infections have been reported in Asia and currently do not represent risks for human health in endemic areas for schistosomiasis (Lee et al., 2017; Kim et al., 2019). However, this cross-reactivity should be taken into account by the local malacological surveillance and schistosomiasis control program to measure the benefits and risks of using the SmMIT-LF-RPA for snail xenomonitoring. Further modifications of the primers and probe could be carried out to prevent the cross-reactivity with this non-*Schistosoma* trematode.

The SmMIT-RPA assay presented a high limit of detection (LOD) being able to detect down to 1 fg of *S*. *mansoni* DNA, with an analytical sensitivity of 20% at this level, but 100% for 1 ng. A reason for the low percentage of analytical sensitivity at low concentrations of *S*. *mansoni* DNA could be due to crowding agents in the RPA assay components. The crowding agents have an important role acting in the formation of the primer-recombinase complex (Piepenburg et al., 2006). However, they can also influence the reaction performance when there are low copies of the target due to its viscosity, which may be the reason for inconsistent results when low amounts of DNA were added to the reaction (Lobato and O’Sullivan, 2018). Moreover, analytical sensitivity does not always correspond to diagnostic sensitivity due to the nature of the starting material. When synthetic copies of the target were used, the LOD was 1 copy with an analytical sensitivity of 100%. This may be due to the higher purity of commercial samples compared to the gDNA dilutions obtained from adult worms’ extracts. In comparison to the LOD of the SmMIT-RPA (down to 1 fg), the SmMIT-LF-RPA presented a 10-fold reduction (down to 10 fg) in the assay’s analytical sensitivity. The difference in sensitivity between LF- and RT-RPA approaches has previously been reported for the assays developed for *S*. *haematobium,* with the LF assay able to detect 100 fg of the gDNA while the RT was able to detect down to 1 fg (Rosser et al., 2015; Rostron et al., 2019).

Both RPAs were able to detect the presence of DNA extracted using the SwiftX™ DNA kit from single *S*. *mansoni* eggs. This kit consists of the same components and steps as the discontinued Speed Xtract Nucleic Acid Kit (Qiagen, Hilden, Germany) and is a simple, fast, and efficient extraction method that requires few laboratory resources (e.g., pipettes and magnetic rack). A modification in the original protocol from the manufacturer was evaluated and the incubation of the sample was tested at both 95°C (as recommended) and at room temperature (~19°C), with the latter allowing to dispense with the requirement for a heating block that may rely on electricity. Both extraction conditions presented positive results. In addition, whole eggs (frozen and fresh) added directly to the reaction mix also produced positive results. This detection of *S*. *mansoni* DNA from single crude eggs reduces the requirements for sample preparation making both SmMIT-RPA and SmMIT-LF-RPA assays even more field-friendly while also reducing costs. However, clinical samples may present additional complications related to processing, egg disruption, and removal of inhibitors particularly related to stool samples. This has been demonstrated for other molecular assays where bead-beating and/or freezing prior to DNA extraction has been shown to increase DNA yields, improving the performance of molecular diagnostics (Pomari et al., 2019; Barda et al., 2020). The use of egg disruption strategies coupled with different DNA extraction methods needs to be further tested for RPA-based assays, particularly for clinical samples.

Recombinase Polymerase Amplification has been tested on multiple types of samples, e.g., urine, stool, blood, bodily fluids, and animal and plant products among others (Daher et al., 2016; Lobato and O’Sullivan, 2018). Moreover, the tolerance of the RPA reaction components to known PCR inhibitors found in clinical samples has been demonstrated in previous studies (Archer et al., 2022), including the direct addition of crude urine into the reaction mix (Rosser et al., 2015). The present study showed that both the LF-and RT-RPA approaches were able to detect *S*. *mansoni* DNA in clinical and spiked samples (stool and urine, respectively). Stool samples are the most used biological material for the molecular detection of *S*. *mansoni*, as it is the source of eggs and cfDNA. Promisingly, both LF-and RT-RPAs developed in this study were able to detect the infection in a stool sample having 1 egg/g that has previously been characterised as positive sample by KK and PCR. Several authors have also reported that urine samples can be used as the source of cfDNA of *S*. *mansoni* (Lodh et al., 2017; Fernández-Soto et al., 2019; Diab et al., 2021; Allam, 2022). In this study, urine samples were spiked with different amounts of gDNA to simulate the detection of cfDNA in clinical samples. As with testing of the gDNA standards, a difference was observed in the sensitivity of the SmMIT-RPA and SmMIT-LF-RPA, with the LF assay being less sensitive when using spiked urine samples. A 10-fold decrease in analytical sensitivity was observed for the LF-versus the RT-RPA assays using gDNA, whereas when the DNA was incorporated into the urine, there was a further loss of sensitivity for the LF assay (1,000-fold decrease). This may result from the RT-RPA having a higher tolerance to the presence of the inhibitors than the LF-RPA. However, additional studies should be conducted with larger numbers of clinical samples to investigate the effect of inhibitors on RPA outcome and whether they need to be removed.

The long-term stability of the lyophilised RT-RPA pellet and reagents has been previously evaluated by Chandu et al. (2016), proving the robustness of the reagents kept at −15–8°C for 1 year, and at 22–28°C for up to 6 months. Our findings confirm the stability of the reagents plus the primers and probe for 3 weeks when stored at 19°C and up to 2 weeks when stored at 27°C, agreeing with results previously obtained by Lillis et al., (2016). Infra-structure limitations in endemic areas may hamper the use of molecular methods. For this reason, the ability to work without dependence of cold chain storage facilitates the use of the SmMIT-RPA in these contexts. The longevity of all reagents for a period longer than 3 weeks or at a temperature higher than 27°C, as well as the impact of humidity, should be evaluated in the future.

Even though RPA presents many advantages in terms of performance and accuracy, the costs of the assay might be a limiting factor considering its application in endemic settings, where usually there is limited availability of financial resources. In general, a qPCR reaction costs 1.5 USD per sample (not including the cost of DNA extraction and the thermocycler; Archer et al., 2022), whereas the KK technique, which is the recommended diagnostic test by WHO and local health authorities, costs approximately 0.1–0.3 USD per sample (not including the cost of microscope and personnel; Speich et al., 2010). The cost of a full-volume SmMIT-RPA per sample is 6.98 USD meanwhile the half-volume reaction cost is 3.49 USD. The SmMIT-LF-RPA has a higher cost per sample given the price of the cassettes, being 9.08 USD for each full-volume reaction, and 5.84 USD, for the half-volume reaction. The half-volume SmMIT-RPA is the most cost-effective protocol among the ones assessed in this study. Though it is 2.3 times more expensive than qPCR, the SmMIT-RPA is simpler and faster, the results are easier to interpret, and it is more field-friendly. The costs of each RPA assay are detailed in Supplementary material 6 and does not include the costs of DNA extractions as well as the cost of the fluorometer and other equipment needed (e.g., pipette, centrifuge, and vortex). The findings from this study represent important progress on price reduction by using half of the reaction volume without compromising good and consistent results for the detection of *S*. *mansoni*. The use of a smaller reaction volume, than that recommended by the manufacturer, has been tested before aiming at the elimination of the mixing step during incubation (Lillis et al., 2016), and this should be tested further with this SmMIT-RPA assay. Further optimisation of the assay and large-scale use may reduce the cost per reaction. Moreover, the cost-benefits of implementing a more sensitive and accurate diagnostic test should be considered. Accurate diagnosis enables timely treatment, the prevention of long-term complications and financial losses due to lack of productivity and sick leaves, as well as reducing the potential of emergence of new transmission foci (Turner et al., 2017). Thus, the impact of using a more expensive diagnostic test will be smaller in the long run given the benefits of its implementation.

The SmMIT-RPA presented a better performance overall when compared to the SmMIT-LF-RPA. Besides being more specific and sensitive, the SmMIT-RPA is advantageous as real-time visualisation of results does not require opening the reaction tubes after DNA amplification. RPA can amplify up to 10^4^-fold the target in 10 min (Daher et al., 2016) and assays requiring end-point visualisation of the result can be prone to cross-contamination among the samples. Therefore, until a closed system is developed and available for the LF assay, there will be a risk of contamination when this approach is used.

Determining the precise prevalence of schistosomiasis in a specific area is conditional to a sensitive, specific, reproducible, and accessible diagnostic method. However, diagnostics improvements are very much needed for detection of light infections in low prevalence settings, and for the verification of transmission interruption (Ogongo et al., 2022; World Health Organization, 2022). If good diagnostic tools are not available, the true prevalence of a specific area may be underestimated limiting the efficiency of schistosomiasis control programs (Panzner, 2022). The promising results obtained in this study suggest that the SmMIT-RPA may allow for a more accurate and rapid diagnosis of schistosomiasis and therefore may influence the decision-making processes involved in determining appropriate destination of public funding aimed at the elimination of schistosomiasis as a public health problem by 2030. Additional analysis will be conducted in order to validate the use of this assay for the detection of *S*. *mansoni* in clinical and field samples from endemic areas.

## Data availability statement

The raw data supporting the conclusions of this article will be made available by the authors, without undue reservation.

## Author contributions

BW, CF, RC, and SM: conceptualization. BW, RC, and SM: methodology and project administration. BW and SM: validation and data curation. SM: formal analysis, writing—original draft, and visualization. BW, EL, and SM: investigation. BW, GM, and RC: resources. BW, CF, EL, GM, and RC: writing—review and editing. BW, CF, and RC: supervision. BW, CF, and SM: funding acquisition. All authors contributed to the article and approved the submitted version.

## Funding

This research has been financed by the Royal Society of Tropical Medicine and Hygiene (RSTMH) via the Small Grant Programme and was partially supported by the Coordenação de Aperfeiçoamento de Pessoal de Nível Superior – CAPES - Finance Code 001; SM is funded by CAPES via the PrINT programme, and Vice-Presidência de Educação, Informação e Comunicação (VPEIC-Fiocruz); CF is funded by CNPq Fellowship (grant number 303131/2018-7); and support was also provided *via* the Natural History Museum’s Departmental Investment Fund and Conselho Nacional de Desenvolvimento Científico/Programa de Excelência em Pesquisa—Pesquisa e Ensaios Clínicos (PROEP/PEC; 420685/2017-0).

## Conflict of interest

The authors declare that the research was conducted in the absence of any commercial or financial relationships that could be construed as a potential conflict of interest.

## Publisher’s note

All claims expressed in this article are solely those of the authors and do not necessarily represent those of their affiliated organizations, or those of the publisher, the editors and the reviewers. Any product that may be evaluated in this article, or claim that may be made by its manufacturer, is not guaranteed or endorsed by the publisher.
