# Supplementary material for: Development of real-time and lateral flow recombinase polymerase amplification assays for rapid detection of Schistosoma mansoni
Source: Front Microbiol. 2022 Nov 18;13:1043596. doi: 10.3389/fmicb.2022.1043596 (PMC9716991; doi:10.3389/fmicb.2022.1043596)
Supplement: Supplementary file 1 [file Table_1.DOCX]

***Supplementary Material 1.* Sample information used within the study.**

| Sample | Isolate info. and origin | Year of collection/isolation | Original Host / Culture | Source |
| --- | --- | --- | --- | --- |
| *Schistosoma haematobium* | Laboratory Culture / Senegal | 1983 | Human / Hamster | Schistosomiasis Collection at The Natural History Museum (SCAN) |
| *Schistosoma curassoni* | Senegal | 2008 | Sheep | Schistosomiasis Collection at The Natural History Museum (SCAN) |
| *Schistosoma bovis* | Senegal | 2008 | Cow | Schistosomiasis Collection at The Natural History Museum (SCAN) |
| *Schistosoma mansoni* | Laboratory culture/ Belo Horizonte-MG, Brazil | 1968 | Human / Laboratory Mice | Helminthology and Medical Malacology Laboratory (HMM) from Fiocruz Minas |
| *Biomphalaria glabrata* | Guadeloupe, France | NA | - | Medical Malacology Collection at René Rachou Institute, Fiocruz Minas (Fiocruz-CMM) |
| *Biomphalaria straminea* | Propriá-SE, Brazil | NA | - | Medical Malacology Collection at René Rachou Institute, Fiocruz Minas (Fiocruz-CMM) |
| *Biomphalaria tenagophila* | Bananal-SP, Brazil | 2010 | - | Medical Malacology Collection at René Rachou Institute, Fiocruz Minas (Fiocruz-CMM) |
| *Ascaris lumbricoides* | Belo Horizonte-MG, Brazil | 1993 | Human | Helminthology and Medical Malacology Laboratory (HMM) from Fiocruz Minas |
| Hookworm | Belo Horizonte-MG, Brazil | 1999 | Human | Helminthology and Medical Malacology Laboratory (HMM) from Fiocruz Minas |
| *Enterobius vermicularis* | Jaboticatubas-MG, Brazil | 2002 | Human | Helminthology and Medical Malacology Laboratory (HMM) from Fiocruz Minas |
| *Trichuris trichiura* | NA | 1993 | Human | Helminthology and Medical Malacology Laboratory (HMM) from Fiocruz Minas |
| *Fasciola hepatica* | Pelotas-RS, Brazil | 2009 | Cattle | Helminthology and Medical Malacology Laboratory (HMM) from Fiocruz Minas |
| Clinostomidae | Belo Horizonte-MG, Brazil | 2014 | *B. glabrata* | Medical Malacology Collection at René Rachou Institute, Fiocruz Minas (Fiocruz-CMM) via the Trematodes Biology Laboratory from Federal University of Minas Gerais |
| Echinostomatidae | Belo Horizonte-MG, Brazil | 2014 | *B. straminea* | Medical Malacology Collection at René Rachou Institute, Fiocruz Minas (Fiocruz-CMM) via the Trematodes Biology Laboratory from Federal University of Minas Gerais |
| Notocotylidae | Belo Horizonte-MG, Brazil | 2014 | *B. straminea* | Medical Malacology Collection at René Rachou Institute, Fiocruz Minas (Fiocruz-CMM) via the Trematodes Biology Laboratory from Federal University of Minas Gerais |
| Spirorchiidae | Belo Horizonte-MG, Brazil | 2014 | *B. straminea* | Medical Malacology Collection at René Rachou Institute, Fiocruz Minas (Fiocruz-CMM) via the Trematodes Biology Laboratory from Federal University of Minas Gerais |
| Strigeidae | Ribeirão das Neves-MG, Brazil | 2015 | *B. glabrata* | Medical Malacology Collection at René Rachou Institute, Fiocruz Minas (Fiocruz-CMM) via the Trematodes Biology Laboratory from Federal University of Minas Gerais |
| Positive stool | Colombia | 2018 | Human | University “Magna Graecia” of Catanzaro |
| Negative stool | Guinea Bissau | 2018 | Human | University “Magna Graecia” of Catanzaro |
| *S. mansoni* eggs | Puerto Rico / Laboratory culture | 2022 | Laboratory Mice | Snail Schistosome Resource (SSR, Natural History Museum, UK) via the NIAID Schistosomiasis Resource Centre (SRC, Biomedical Research Institute, USA) |

Legend: NA- not available; SE- Brazilian state of Sergipe; SP- Brazilian state of São Paulo; MG- Brazilian state of Minas Gerais; RS- Brazilian state of Rio Grande do Sul.
