# Supplementary material for: Development of real-time and lateral flow recombinase polymerase amplification assays for rapid detection of Schistosoma mansoni
Source: Front Microbiol. 2022 Nov 18;13:1043596. doi: 10.3389/fmicb.2022.1043596 (PMC9716991; doi:10.3389/fmicb.2022.1043596)
Supplement: Supplementary file 2 [file Table_2.DOCX]

***Supplementary Material 2.* RPA protocol of the developed assays.**

1. **SmMIT-RPA (real-time fluorescence-based assay)**
2. Prepare a Master Mix (MM) in a DNA-free area

- For each sample used, add in a 2ml tube:
- 2.1µL forward primer
- 2.1µL reverse primer
- 0.6µL fluorescent probe
- 29.5µL rehydration buffer
- Add 34.3 µL to the RPA pellet and homogenise
- For half-volume reactions, transfer 17.15µL from the “MM+pellet” to a new tube
- For full-volume reactions, add 2.5µL of MgAc into the tube’s lid or,
- For half-volume reactions, add 1.25µL of MgAc into the tube’s lid
- Close the tube carefully and transfer to the DNA area.

1. Add the DNA and/or water until completing 50µL (full-volume reactions) or 25µL (half-volume reactions)
2. Mix by inversion
3. Quickly centrifuge (~2s)
4. Immediately place the tubes inside the fluorometer
5. Incubate at 42°C for 4 minutes
6. Mix by inversion
7. Quickly centrifuge (~2s)
8. Immediately place the tubes inside the fluorometer

10. Incubate at 42°C until complete 20 minutes

11. Check the result on the fluorometer touchscreen or export the raw data on a USB for further analysis

**B. SmMIT-LF-RPA (lateral flow assay)**

1. Prepare a Master Mix (MM) in a DNA-free area

- For each sample used, add in a 2ml tube:
- 2.1µL forward primer
- 2.1µL reverse primer
- 0.6µL fluorescent probe
- 29.5µL rehydration buffer
- Add 34.3 µL to the RPA pellet and homogenise
- For half-volume reactions, transfer 17.15µL from the “MM+pellet” to a new tube
- For full-volume reactions, add 2.5µL of MgAc into the tube’s lid or,
- For half-volume reactions, add 1.25µL of MgAc into the tube’s lid
- Close the tube carefully and transfer to the DNA area.

1. Add the DNA and/or water until completing 50µL (full-volume reactions) or 25µL (half-volume reactions)
2. Mix by inversion
3. Quickly centrifuge (~2s)
4. Immediately place the tubes at 42°C
5. Incubate at 42°C for 4 minutes
6. Mix by inversion
7. Quickly centrifuge (~2s)

9. Incubate at 42°C until complete 20 minutes

10. Prepare a mix containing 5 µL of the amplification product and 70µL of the PCRD buffer and homogenise

11. Transfer 75µL of the mixture to the PCRD cassettes

12. Wait for 10 minutes

13. Check the result

- Control line only: negative result

- Control and test lines: positive result

- Test line only: invalid result
