# Supplementary material for: Development of real-time and lateral flow recombinase polymerase amplification assays for rapid detection of Schistosoma mansoni
Source: Front Microbiol. 2022 Nov 18;13:1043596. doi: 10.3389/fmicb.2022.1043596 (PMC9716991; doi:10.3389/fmicb.2022.1043596)
Supplement: Supplementary file 3 [file Table_3.DOCX]

***Supplementary Material 3. Schistosoma mansoni* eggs isolation and DNA extraction.**

Single eggs were isolated and directly added to the reaction mix which was either kept frozen or in fresh condition. Single eggs were also isolated for DNA extraction using the SwiftX™ DNA extraction kit. Images used in this figure were obtained at Bioicons and SMART- Servier Medical Art websites.

**
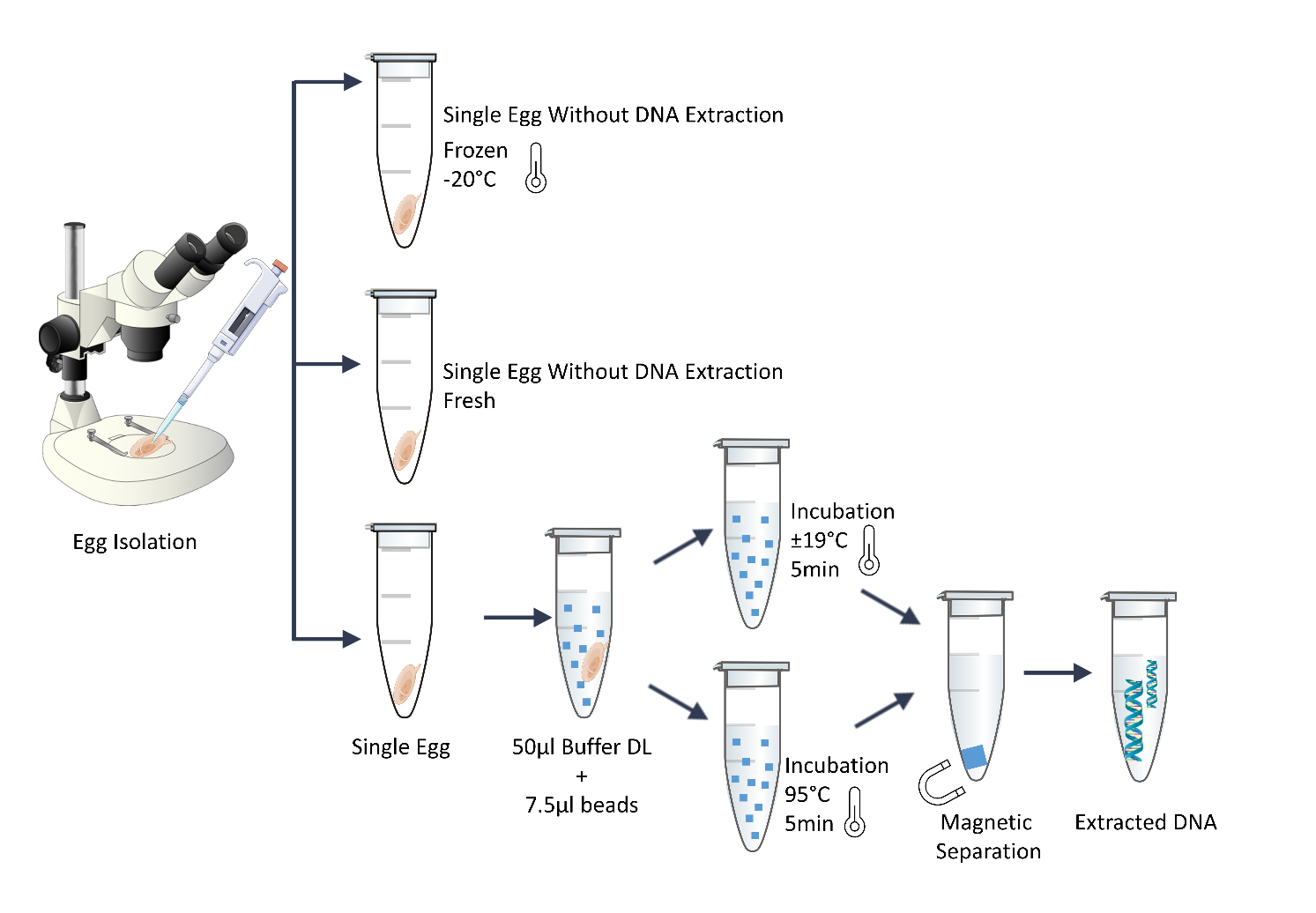
**
