# Supplementary material for: Development of real-time and lateral flow recombinase polymerase amplification assays for rapid detection of Schistosoma mansoni
Source: Front Microbiol. 2022 Nov 18;13:1043596. doi: 10.3389/fmicb.2022.1043596 (PMC9716991; doi:10.3389/fmicb.2022.1043596)
Supplement: Supplementary file 4 [file Table_4.DOCX]

***Supplementary Material 4.* Real-time and lateral-flow RPAs performed using full and half-volume reactions.**

**
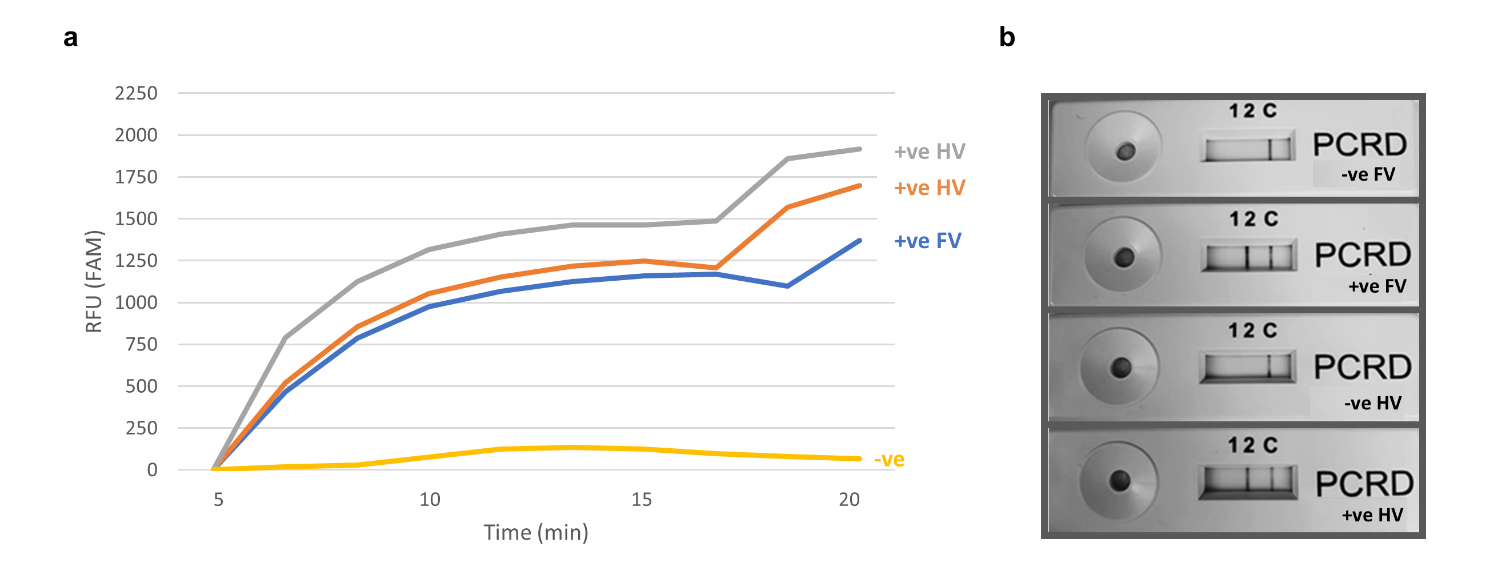
**

**a.** Using full and half volume of the TwistAmp® Exo kit to perform the SmMIT-RPA; and **b.** using full and half volume of the TwistAmp® Nfo kit to perform the SmMIT-LF-RPA. Graph a. is displayed with the background baseline fluorescence subtracted.

Legend: RFU- relative fluorescence units; +ve- positive control (1 ng of *S. mansoni* gDNA); -ve- negative control (water), FV- full-volume reaction; HV- half-volume reaction.
