# Supplementary material for: Development of real-time and lateral flow recombinase polymerase amplification assays for rapid detection of Schistosoma mansoni
Source: Front Microbiol. 2022 Nov 18;13:1043596. doi: 10.3389/fmicb.2022.1043596 (PMC9716991; doi:10.3389/fmicb.2022.1043596)
Supplement: Supplementary file 5 [file Table_5.DOCX]

***Supplementary Material 5.* Laboratory validation of the RPA assays using biological samples.**

1. **Laboratory validation of SmMIT-RPA**


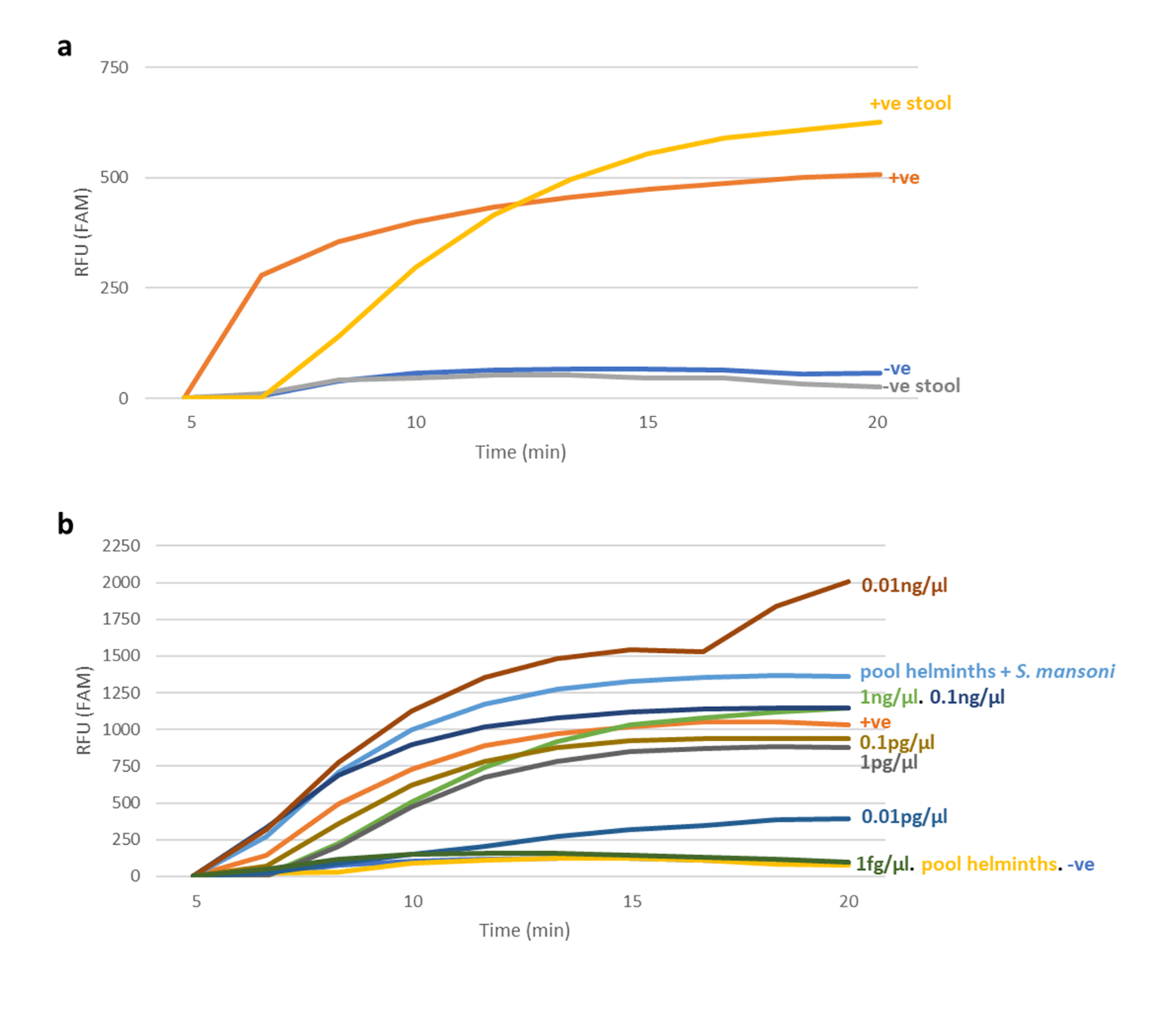


**a.** *S. mansoni* positive stool sample (1 egg/g) and, **b.** urine samples spiked with down to 10 fg/µl of *S. mansoni* DNA. All graphs are displayed with the background baseline fluorescence subtracted.

Legend: RFU- relative fluorescence units; +ve- positive control (1 ng of *S. mansoni* gDNA); -ve- negative control (water); ng- nanogram; pg- picogram; fg- femtogram.

1. **Laboratory validation of the SmMIT-LF-RPA**


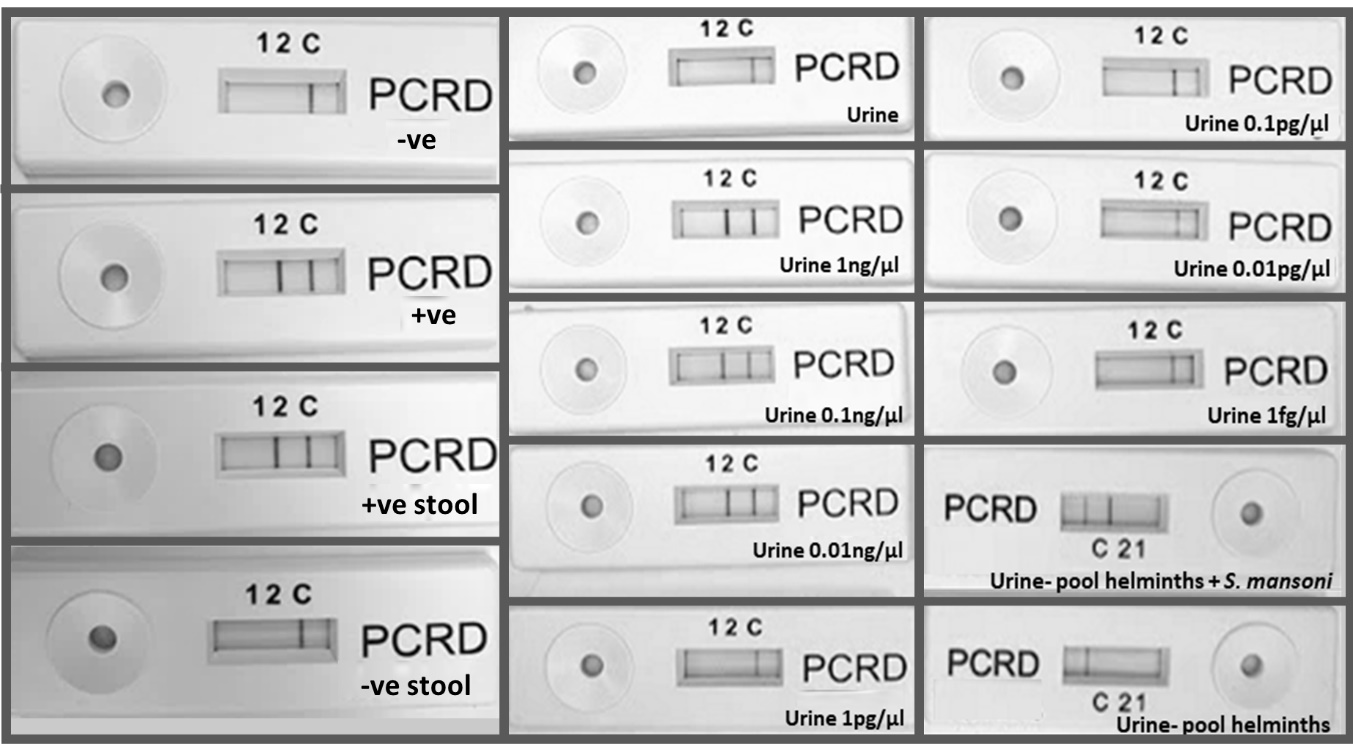


The assay was positive when tested on DNA from the stool sample containing one *S. mansoni* egg /g of stool and in spiked urine samples (10 pg/µl of *S. mansoni* DNA).

Legend: ng- nanogram; pg- picogram, fg- femtogram; +ve- positive control (1 ng of *S. mansoni* gDNA); -ve- negative control (water).

.
