# Supplementary material for: Development of real-time and lateral flow recombinase polymerase amplification assays for rapid detection of Schistosoma mansoni
Source: Front Microbiol. 2022 Nov 18;13:1043596. doi: 10.3389/fmicb.2022.1043596 (PMC9716991; doi:10.3389/fmicb.2022.1043596)
Supplement: Supplementary file 6 [file Table_6.DOCX]

***Supplementary Material 6.* Costs of the RPA assays developed.**

| Reagent | Manufacturer | Assay | Cost*  (USD) | No. of reactions | | Reagent cost/sample | | Assay cost/sample | |
| --- | --- | --- | --- | --- | --- | --- | --- | --- | --- |
|  |  |  |  | **Full-volume** | **Half-Volume** | **Full-volume** | **Half-Volume** | **Full-volume** | **Half-Volume** |
| TwistAmp Exo Kit | TwistDX | RT | 284.52 | 96 | 192 | 2.96 | 1.48 | 6.98 | 3.49 |
| Primer Forward | TIB MolBio | RT | 25.80 | 619 | 1238 | 0.04 | 0.02 |  |  |
| Primer Reverse | TIB MolBio | RT | 25.80 | 576 | 1152 | 0.04 | 0.02 |  |  |
| Exo Probe | TIB MolBio | RT | 353.61 | 90 | 180 | 3.93 | 1.96 |  |  |
| TwistAmp Nfo Kit | TwistDX | LF | 284.52 | 96 | 192 | 2.96 | 1.48 | 9.08 | 5.84 |
| Primer Forward | TIB MolBio | LF | 25.8 | 619 | 1238 | 0.04 | 0.02 |  |  |
| Primer Reverse | TIB MolBio | LF | 92.13 | 147 | 295 | 0.63 | 0.31 |  |  |
| Nfo Probe | TIB MolBio | LF | 256.03 | 90 | 180 | 2.84 | 1.42 |  |  |
| PCRD Kit | Abingdon Health | LF | 130.11 | 50 | 50 | 2.60 | 2.60 |  |  |

*****The costs of each reagent were calculated according to the US Dollar exchange rate for 15/07/2022 from the Bank of England (<https://www.bankofengland.co.uk/>) when 1 GBP= 1.1855 USD and 1 EUR= 1.008 USD.
